# Recurrence pattern analysis after re-irradiation with bevacizumab in recurrent malignant glioma patients

**DOI:** 10.1186/s13014-014-0299-y

**Published:** 2014-12-21

**Authors:** Maximilian Niyazi, Nathalie Lisa Jansen, Maya Rottler, Ute Ganswindt, Claus Belka

**Affiliations:** Department of Radiation Oncology, University Hospital of Munich, Marchioninistr. 15, 81377 Munich, Germany; Department of Nuclear Medicine, University of Munich, Marchioninistr. 15, 81377 Munich, Germany

**Keywords:** Recurrence pattern, Re-irradiation, Bevacizumab, Glioblastoma

## Abstract

**Background:**

The aim of the present analysis was to evaluate the recurrence pattern in patients with recurrent malignant glioma after re-irradiation in combination with bevacizumab as there is limited data on how to optimally choose dose, fractionation and delineation margins.

**Methods:**

Thirty-one patients with recurrent malignant glioma treated with re-irradiation and bevacizumab after previous chemoradiotherapy (concurrent temozolomide 75 mg/m^2^/d according to the EORTC/NCIC trial) and [^18^ F]FET-PET and/or MRI confirmed recurrence were retrospectively analyzed. Bevacizumab was applied twice during fractionated re-irradiation (10 mg/kg, d1 + d15, median 36 Gy, conventionally fractionated). Recurrence patterns were assessed by means of [^18^ F]FET-PET and/or MRI.

**Results:**

Median follow-up was 34.0 months for all patients [95%-CI, 27.7-40.3] and median post-recurrence survival 10.8 months [95%-CI, 9.2-12.4]. Concerning the recurrence patterns, 61.3% of these were located in-field (19 patients), 22.6% were marginal (7 patients) and 16.1% ex-field (5 patients). No influence on the recurrence pattern was observed according to sex, WHO grade, maintenance chemotherapy or MGMT methylation status whereas planning target volume (PTV) size had a significant influence on the recurrence pattern (p = 0.032). PTV sizes > 75 ml were associated with a higher in-field recurrence rate and lower median post-recurrence progression-free survival (8.5 vs. 4.9 months, p = 0.016).

**Conclusions:**

After the administration of re-irradiation with bevacizumab the recurrence pattern seems to be mainly centrally located. The PTV size was the main predictor for a marginal/ex-field recurrence.

## Introduction

In patients with high-grade glioma (HGG) a high rate of local failures has been observed after multimodal therapy [1]. The addition of temozolomide (TMZ) increased local control and survival whereas the 2-year survival rate remained 27.2% [2].

In selected patients, a second course of radiotherapy (RT) might be a reasonable treatment option despite the relative lack of prospective randomized data [3-6]. Contrarily, conventional cytotoxic approaches were found to be not adequately effective [7-10] so molecularly targeted drugs either alone or in combination with cytotoxic agents are currently undergoing clinical testing.

Various groups have investigated the use of bevacizumab – a humanised monoclonal antibody against VEGF-A with an already established role in metastatic colon, breast, and lung cancer [11] – for patients with recurrent HGG [12] and several trials have documented its efficacy [13-17] which may be due to the presence of pronounced hypoxia as well as high levels of tumor driven angiogenesis in HGG [18].

Since the efficacy of radiation-based re-treatment is limited, it is reasonable to test how far the addition of a putative radiation response modulator would impact on the efficacy of re-treatment. In this regard, one group tested the sequential use of radiosurgery and bevacizumab with favorable outcome [19]. Alternatively, Gutin and co-workers determined the safety and efficacy of radiotherapy (RT) and concomitant bevacizumab – for the GBM cohort, progression-free survival at six months (PFS-6) was 65% [20]. In a previous retrospective study on 30 recurrent malignant glioma patients undergoing re-irradiation, 20 treated with bevacizumab and 10 without bevacizumab we showed that PFS-6 within the bevacizumab-treated cohort was 72% and survival was significantly enhanced [21]. With substantially longer follow-up and a higher patient number, the significant post-recurrence survival (PRS) benefit of bevacizumab could be confirmed within a second study describing a beneficial treatment with a low rate of side-effects [22]. Recent prospective phase III trials (AVAglio & RTOG 0825) were designed to prove the efficacy of TMZ based radio-chemotherapy with bevacizumab as first-line therapy but failed to show a survival benefit whereas significant and marginally significant progression-free survival benefits have been observed [23,24].

In our study we retrospectively analyzed the pattern of re-recurrence in recurrent HGG patients undergoing re-irradiation with bevacizumab as there is limited data on how to choose proper safety margins during radiotherapy planning and furthermore, in how far the chosen fractionation schedule yielded adequate local control rates.

## Material and Methods

### Patient selection

Only patients with histologically and/or [^18^ F]FET-PET/MRI proven recurrence of high-grade gliomas (WHO grades III + IV) and macroscopic tumor (maximum diameter 6 cm, multifocality per se was not a contraindication) underwent re-irradiation, the interval between first radiotherapy and re-irradiation had to be 6 months at minimum. Another precondition was the absence of meaningful alternative treatment options, e. g. complete resection by re-surgery, interstitial brachytherapy or systemic chemotherapy.

### Treatment schedule and follow-up

Before treatment, a gadolinium-enhanced brain MRI with gradient echo sequence and perfusion and/or a [^18^ F]FET-PET. Patients treated with bevacizumab received 10 mg/kg at days 1 and 15 during re-irradiation. If TMZ was applied concomitantly in patients who had no previous progression after TMZ pre-treatment a dosage of 75 mg/m^2^ daily was chosen.

Treatment outcome was evaluated on a regular basis (every three months) by brain MRI as described by [25] and/or [^18^ F]FET-PET.

Adjuvant chemotherapy was prescribed on an individual basis as no standard has been defined yet but was not defined as mandatory.

### [^18^ F]FET-PET data acquisition and analysis

Dynamic PET scans were acquired on a Siemens ECAT EXACT HR+ scanner (Siemens/CTI, Knoxville, TN, USA) after intravenous injection of approximately 180 MBq [^18^ F]FET according to a standardized protocol [26]. Data were reconstructed by filtered back projection using a Hann filter, corrected for scatter and attenuation and afterwards transferred to a HERMES workstation (Hermes Medical Solutions, Sweden) for further data processing. PET-based evaluation of recurrence was performed by an experienced nuclear physician by assessment of the maximal standardized uptake value within the tumour corrected for the unspecific uptake in the background (SUV_max_/BG > 1.6) in combination with a previously introduced dynamic analysis of [^18^ F]FET kinetic uptake behaviour [27]. Furthermore, the biological tumor volume was assessed, which was defined by semi-automatic threshold-based calculation of a volume of interest.

### Radiotherapy

By analogy with Combs et al. [28] patients received a total dose of 36 Gy in 18 fractions (2 Gy single doses) employing 3D conformal radiotherapy or intensity-modulated radiotherapy (IMRT) if adjacent critical structures were present. Planning target volume (PTV) was defined as gross tumor volume (GTV) plus 10 mm margin at maximum. GTV included the contrast enhancing lesion in T1w + Gd MRI. To ensure reproducibility patients were immobilized with a thermoplastic mask system. Treatment planning was performed using the Oncentra® treatment planning system (OTP MasterPlan®, Elekta, Crawley, UK).

### Analysis of recurrence pattern

Recurrences were defined as “in-field” if more than 80% of the tumour recurrence resided within the prescription 95% isodose surface, and “marginal” if 20% to 80% of the lesion was inside the 95% isodose surface. In all other cases, recurrences were defined as outside the radiation field (“ex-field”) according to the study of Lee et al. [29]; in case of a multifocal recurrence, the part lying most distant to the initial tumour site was taken as reference.

### Statistics

Tumour progression was defined according RANO criteria [30] or by the appearance of new vital/progressive tumour lesions by means of dynamic [^18^ F]FET-PET. Post-recurrence progression-free (PR-PFS) and post-recurrence survival (PRS), measured from the beginning of re-irradiation to progression or death, respectively, or date of last follow-up, were analyzed using the Kaplan-Meier method; 95% CIs were calculated using the associated estimated standard errors. The log-rank test was used to test the significance of the following prognostic variables: MGMT promoter methylation status, age, surgery and pattern of disease recurrence. In view of their small number, patients with recurrence at RT margin were grouped with patients having an ex-field recurrence in the analysis of time to progression. A logistic regression analysis was performed to determine variables with significant influence on the recurrence pattern. Proportions were compared using Fisher’s exact test. A p-value ≤ 0.05 was considered significant.

The scientific use of retrospective data has been explicitly allowed by Bavarian federal law. Additionally, all patients agreed that their scientific data could be used. No experimental research on humans or animals has been performed or reported. The declaration of Helsinki has been obeyed in all points.

## Results

Thirty-one patients with recurrent HGG were included into this retrospective analysis and treated at the Department of Radiation Oncology, University Hospital of Munich from 8/2008 until 7/2012, median age was 51 years (range, 18 – 67 years) and 74.2% of the patients were younger than 60 years.

Median radiotherapy dose was 36 Gy in 2 Gy single fractions and two applications of bevacizumab had been applied concomitantly.

MGMT methylation status was not available in 2 cases (6.5%), 11 of the remaining 29 patients were MGMT methylated (35.5%), for other patient characteristics see Table 1.

**Table 1:** **Patient characteristics, N = 31**

**Characteristic**	**Patients**
Sex	
• Male	21 (67.7%)
• Female	10 (32.3%)
Median Age [y]	51.0 (18 – 67)
Median KPS	80 (40 – 100)
• KPS < 70	8 (25.8%)
• KPS ≥ 70	23 (74.2%)
Median dose of primary radiotherapy	60 Gy
Median dose of re-irradiation	36 Gy
Median PTV size [ml]	118.1 (34.3 – 363.9)
MGMT methylation status	
• methylated	11 (35.5%)
• not methylated	18 (58.1%)
• unknown	2 (6.5%)
WHO grade at relapse	
• III	6 (19.4%)
• IV	25 (80.6%)
Recurrence pattern	
• in-field	19 (61.3%)
• marginal	7 (22.6%)
• distant	5 (16.1%)
Imaging type for recurrence	
• MRI/CT	10 (32.3%)
• PET	21 (67.7%)

Median follow-up was 34.0 months for all patients [95%-CI, 27.7 - 40.3], median post-recurrence survival was 10.8 months [95%-CI, 9.2 - 12.4].

Concerning the recurrence patterns of the 31 patients, 61.3% of these were located in-field (19 patients), 22.6% were marginal (7 patients) and 16.1% ex-field (5 patients).

In view of the small numbers, we summarized both patients with marginal and ex-field recurrences as these were patients where a 10 mm PTV margin was probably too narrow (38.7%). Thus, marginal and ex-field recurrences were grouped and subsequent analyses were based on this precondition.

Concerning PRS and PR-PFS, no significant univariate factors could be obtained with an influence on latter endpoints including KPS, sex, age, WHO grade at relapse, MGMT methylation status, PTV size (continuous variable) or recurrence patterns.

Therefore, we further analyzed which factors would influence the pattern of recurrence.

Univariate testing was performed employing the same factors without the KPS - all could be identified to be non-significant except the PTV size with a hazard ratio of 0.986 (p = 0.032), see Table 2.

**Table 2:** **Logistic regression analysis on recurrence pattern (in-field vs. marginal/distant), N = 31, ns – not significant, meth – MGMT methylated, HR – h** **azard ratio**

**Variable**	**Univariate p-value**	**HR**
**Age (<60 y, ≥ 60 y)**	ns (p = 0.423)	1.875
**MGMT (meth/not meth)**	ns (p = 0.361)	2.00
**PTV size**	p = 0.032	0.986
**WHO grade at relapse (III/IV)**	ns (p = 0.165)	0.200
**Sex (male/female)**	ns (p = 0.914)	0. 923

We performed an ROC analysis to determine the optimal threshold of the PTV to stratify between in-field and marginal/ex-field recurrences and determined 75 ml as optimal size (which is equal to the 25th quartile of all PTV sizes).

Fisher’s exact test showed a trend for a correlation between recurrence pattern and PTV size (as binary variable with 75 ml as threshold); 5 recurrences were marginal/ex-field compared to 2 in-field recurrences for PTV sizes smaller than 75 ml whereas larger PTV sizes were associated with a high in-field recurrence rate, 17 in-field vs. 7 ex-field recurrences (p = 0.078). This constructed binary variable showed a trend in predicting the type of recurrence (hazard ratio 0.17, p = 0.058).

**Figure 1:**
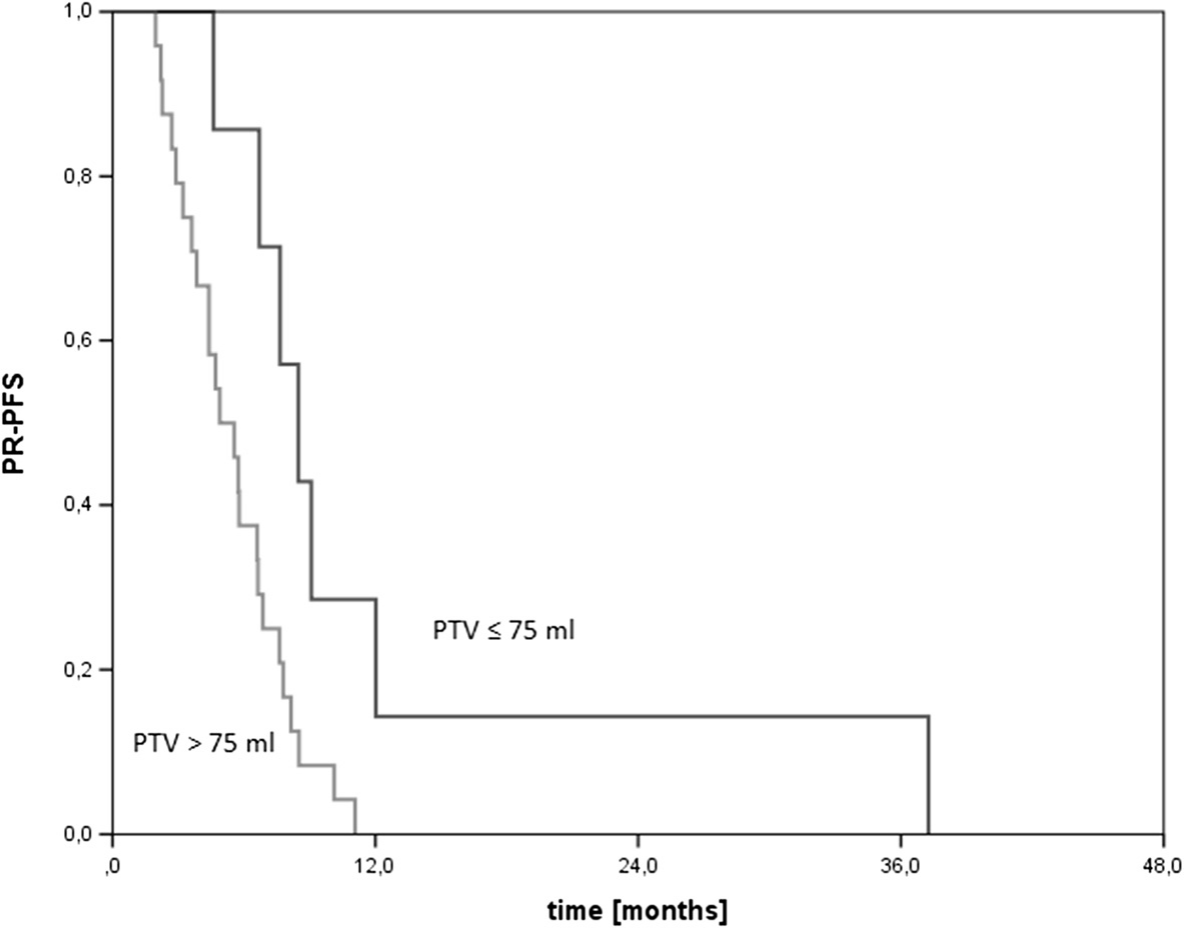
**PR-PFS for patients with PTV size > 75 ml or ≤ 75 ml.**

Finally, we compared the PFS rates in both PTV size groups and obtained a significant difference (8.5 vs. 4.9 months, p = 0.016) which is shown in Figure 1. PRS was not significantly different in both groups (11.3 vs. 10.1 months, p = 0.276).

## Discussion

For certain subgroups of recurrent high-grade glioma patients re-irradiation may be a strategy to prolong survival with acceptable toxicity. The aim of this study was to analyze the re-recurrence pattern after re-irradiation keeping in mind that this is the largest cohort to our knowledge uniformly treated with re-irradiation and bevacizumab in one center.

A mainstay in treatment follow-up is a reliable imaging modality: Whereas conventional magnetic-resonance-imaging (MRI) provides information on the size and localization of the tumor and delineates secondary phenomena such as haemorrhage, oedema, and mass effect, positron emission tomography (PET) with diverse radiolabelled compounds has been proposed to characterize the tumour on a metabolic and molecular level [31]. In particular, different radiolabelled amino acids like [^11^C]Methionine ([^11^C]MET) [32] as well as aromatic amino acid analogues like [^18^ F]Fluoroethyltyrosine ([^18^ F]FET) [33] were previously proposed to provide valuable additional information in patients with glioma [34,35].

MRI was regularly performed as follow-up imaging - [^18^ F]FET-PET imaging was routinely performed (67.7%) or in case of a questionable progression (according to MRI), as it was proposed to reliably distinguish between post-therapeutic benign lesions and tumour recurrence after treatment of low- and high-grade gliomas [36,37].

To our knowledge, the only group having also determined the recurrence pattern after re-irradiation with bevacizumab were Shapiro et al. [38] who derived 50% central recurrences. Their treatment strategy was built on a very tight margin with 5 mm to the contrast-enhancing lesion including 5 fractions with 6 Gy single doses, two fractions per week.

From our observation, 2/36 Gy is locally active in combination with bevacizumab, but recurrences mainly seem to be central which leaves us to speculate that a dose escalation could be warranted - especially due to the radioprotective potential of bevacizumab [39]. Other groups have tested 36 Gy in 2 Gy single fractions with adequate to very moderate activity, recent and several ongoing studies include rather higher single doses (2.4 Gy, 3 Gy, 3.5 Gy).

For smaller lesions, marginal and ex-field recurrences are more often observed which could generate the hypothesis that larger lesions display an increased radioresistant behavior, potentially due to hypoxic conditions and a larger number of tumor stem cells. These findings might lead to an adaption of planning margins according to the size of the lesion, e. g. with a central simultaneous boost technique in large tumor volumes which might be justified by the altogether dismal prognosis of this patient group. As target delineation is mainly based on contrast-enhanced MRI, the relatively higher rate of ex-field recurrences in smaller lesions could be explained by a tumor miss due to small margins (whereas 10 mm are at the higher end of the literature).

The discussion about the tumor size itself as prognostic parameter has not been resolved - despite its relevance on the recurrence pattern, there was no significant influence on survival - even the prognostic score defined by Combs et al. did not include the size itself as parameter but could itself not be validated in smaller cohorts up to now [40-42].

Other recurrence analyses in the primary setting have determined a relation with molecular genetics [1,43] which could not be confirmed in the recurrence setting. Furthermore, no increase in distant failures could be observed such as in smaller series of primary glioblastoma patients after concurrent radiotherapy, bevacizumab and temozolomide [44]. Conversely, the low rate of in-field recurrences was not reproducible due to dose limitations during re-irradiation.

All in all, after the administration of re-irradiation with bevacizumab recurrences seem to be mainly centrally located. The PTV size was the main predictor for a marginal/ex-field recurrence. It would be valuable to have future studies evaluating the role of dose escalation to the central part of the tumor, e. g. as a stereotactic dose escalation or by means of a simultaneous integrated boost (SIB) as shown for many other sites [45-47].
